# Author Correction: A BDNF-TrkB autocrine loop enhances senescent cell viability

**DOI:** 10.1038/s41467-022-35154-z

**Published:** 2022-12-07

**Authors:** Carlos Anerillas, Allison B. Herman, Rachel Munk, Amanda Garrido, Kwan-Wood Gabriel Lam, Matthew J. Payea, Martina Rossi, Dimitrios Tsitsipatis, Jennifer L. Martindale, Yulan Piao, Krystyna Mazan-Mamczarz, Jinshui Fan, Chang-Yi Cui, Supriyo De, Kotb Abdelmohsen, Rafael de Cabo, Myriam Gorospe

**Affiliations:** 1 Laboratory of Genetics and Genomics, National Institute on Aging Intramural Research Program, National Institutes of Health, Baltimore, MD USA; 2 Translational Gerontology Branch, National Institute on Aging Intramural Research Program, National Institutes of Health, Baltimore, MD USA

**Keywords:** DNA damage response, Senescence, Ageing

Correction to: *Nature Communications* 10.1038/s41467-022-33709-8, published online 20 October 2022

The original version of this Article contained an error in Fig. 7h, in which the panel for kidney was inadvertently duplicated and presented both for the kidney and liver. The source data file was not affected by the error, and contains the correct values for both the kidney and liver. The error has been corrected in both the PDF and HTML versions of the Article.

