# Supplementary material for: A BDNF-TrkB autocrine loop enhances senescent cell viability
Source: Nat Commun. 2022 Oct 20;13:6228. doi: 10.1038/s41467-022-33709-8 (PMC9585019; doi:10.1038/s41467-022-33709-8)
Supplement: Supplementary file 1 — Supplementary Information [file 41467_2022_33709_MOESM1_ESM.pdf]

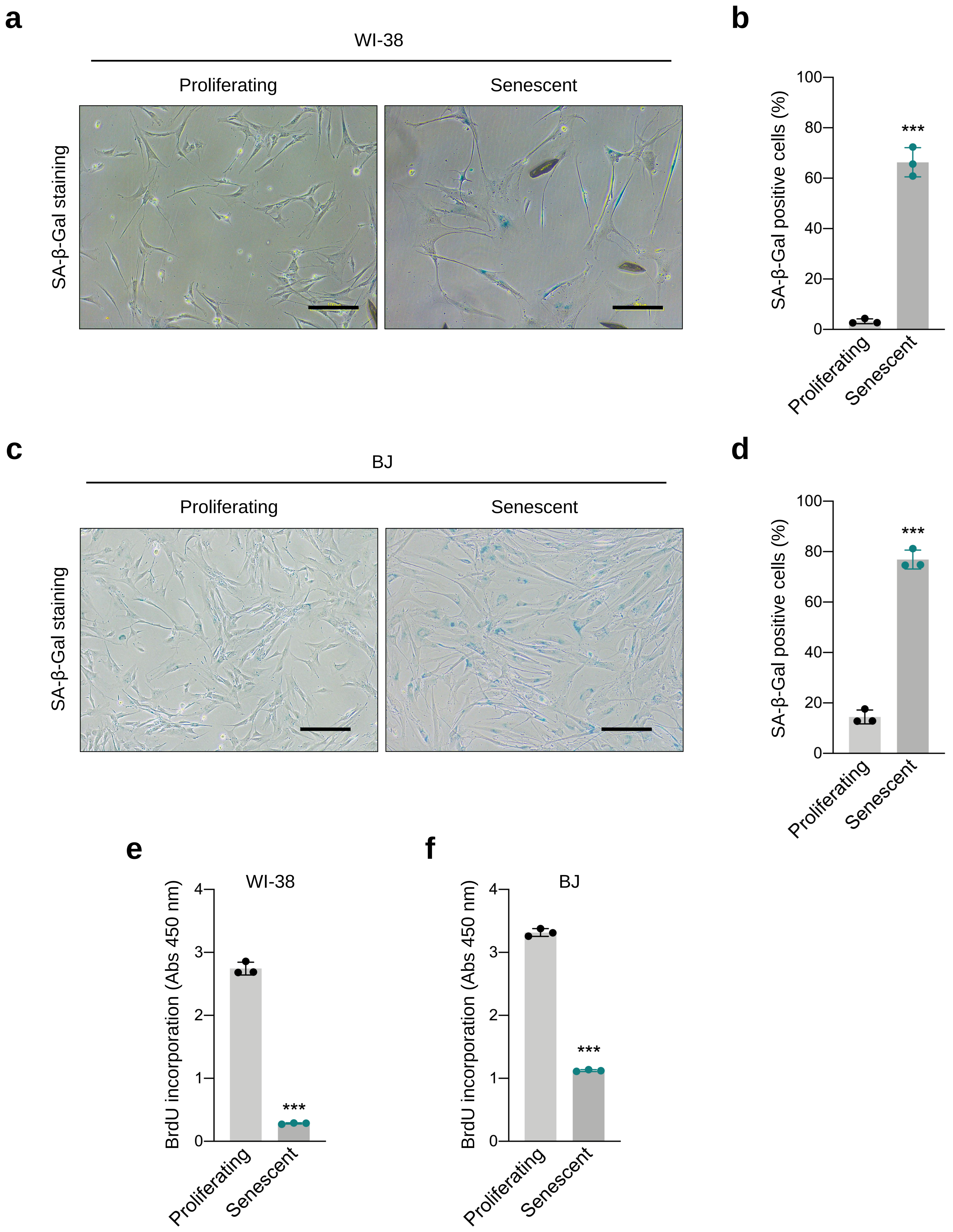

Supplementary Fig. 1. Extended data on the implementation of senescence in WI-38 and BJ fibroblasts. **a** Representative micrographs of SA-β-Gal staining performed in proliferating and senescent WI-38 fibroblasts. Senescence was induced by treating cells with 50 µM etoposide (Etop) for 10 days. Scale bars, 100 µm. **b** Quantification of SA-β-Gal-positive cells in the conditions described in (a). **c** Representative micrographs of SA-β-Gal staining performed in proliferating and senescent BJ fibroblasts. Senescence was induced by treating cells with 25 µM etoposide for 10 days. Scale bars, 100 µm. **d** Quantification of SA-β-Gal-positive cells in the conditions described in (c). **e, f** Evaluation of the proliferative ability for the conditions described in (a) and (c), respectively, by measuring BrdU incorporation 24 h later in WI-38 (e) and BJ (f) fibroblasts. Graphs in b, d, e, and f represent the mean values ±SD of n=3 experiments.

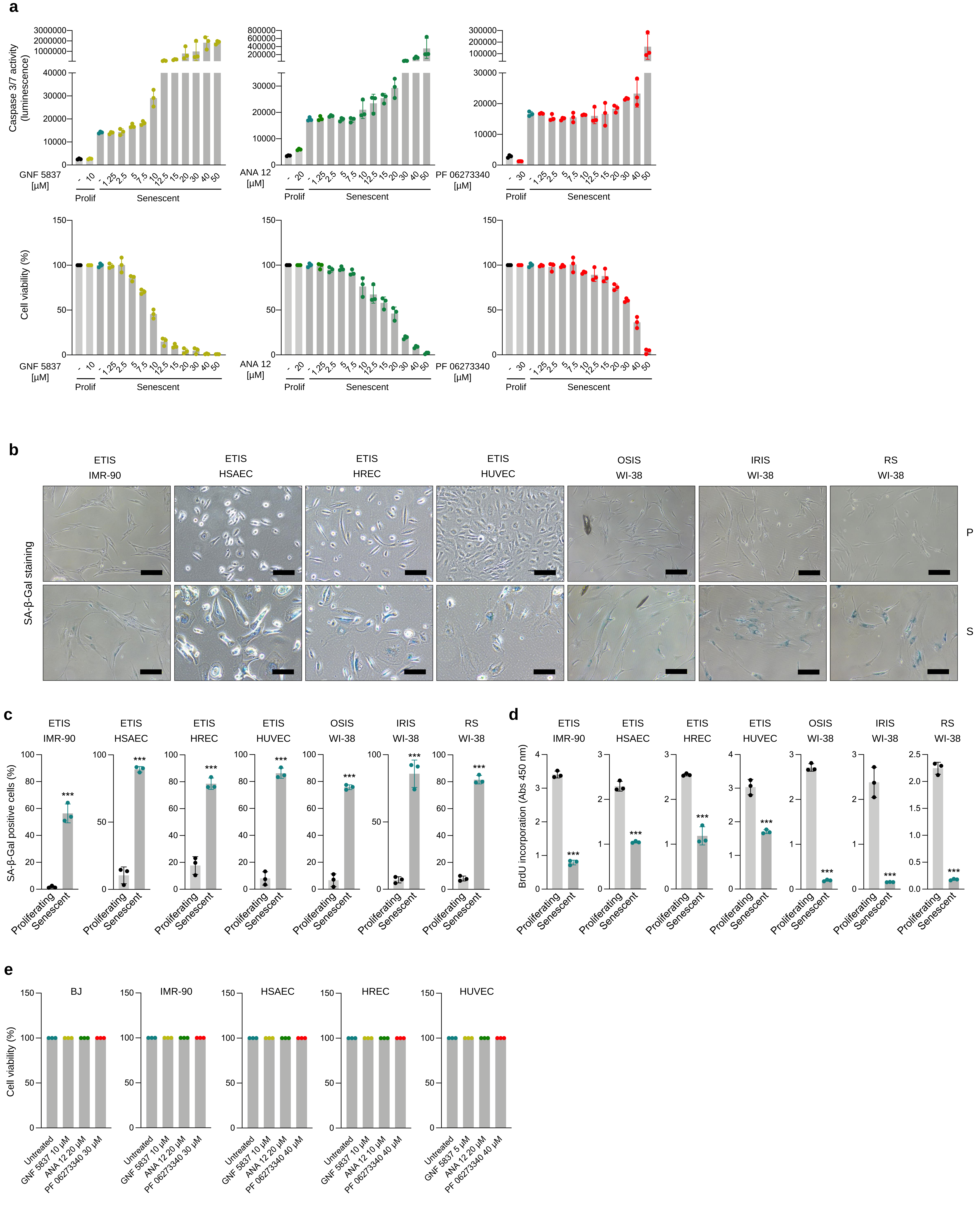

Supplementary Fig. 2. Extended data on Trk inhibitors as senolytic compounds. **a** Assessment of caspase 3/7 activity levels (*top*) and cell viability (*bottom*) 48 h after addition of the indicated doses of Trk inhibitors GNF 5837 (yellow), ANA 12 (green), PF 06273340 (red) in proliferating or etoposide-induced senescent WI-38 cells. **b** Representative images of SA-β-Gal staining performed in proliferating and senescent different cell types indicated in the figure (etoposide-induced senescence, ETIS; oxidative stress-induced senescence, OSIS; ionizing radiation-induced senescence, IRIS; replicative senescence, RS). Scale bars, 100 µm. **c** Quantification of the conditions described in (b). **d** Analysis of the proliferative ability of the experimental conditions described in (b) by BrdU incorporation assay. **e** Cell viability observed after treatment of proliferating control cells from the models described in (b) with the indicated doses of TrkB inhibitors. Graphs in a, and c-e represent the mean values ±SD of n=3 experiments; significance (*p < 0.05, **p < 0.01, ***p < 0.001) was determined by using two-tailed Student's t-test.

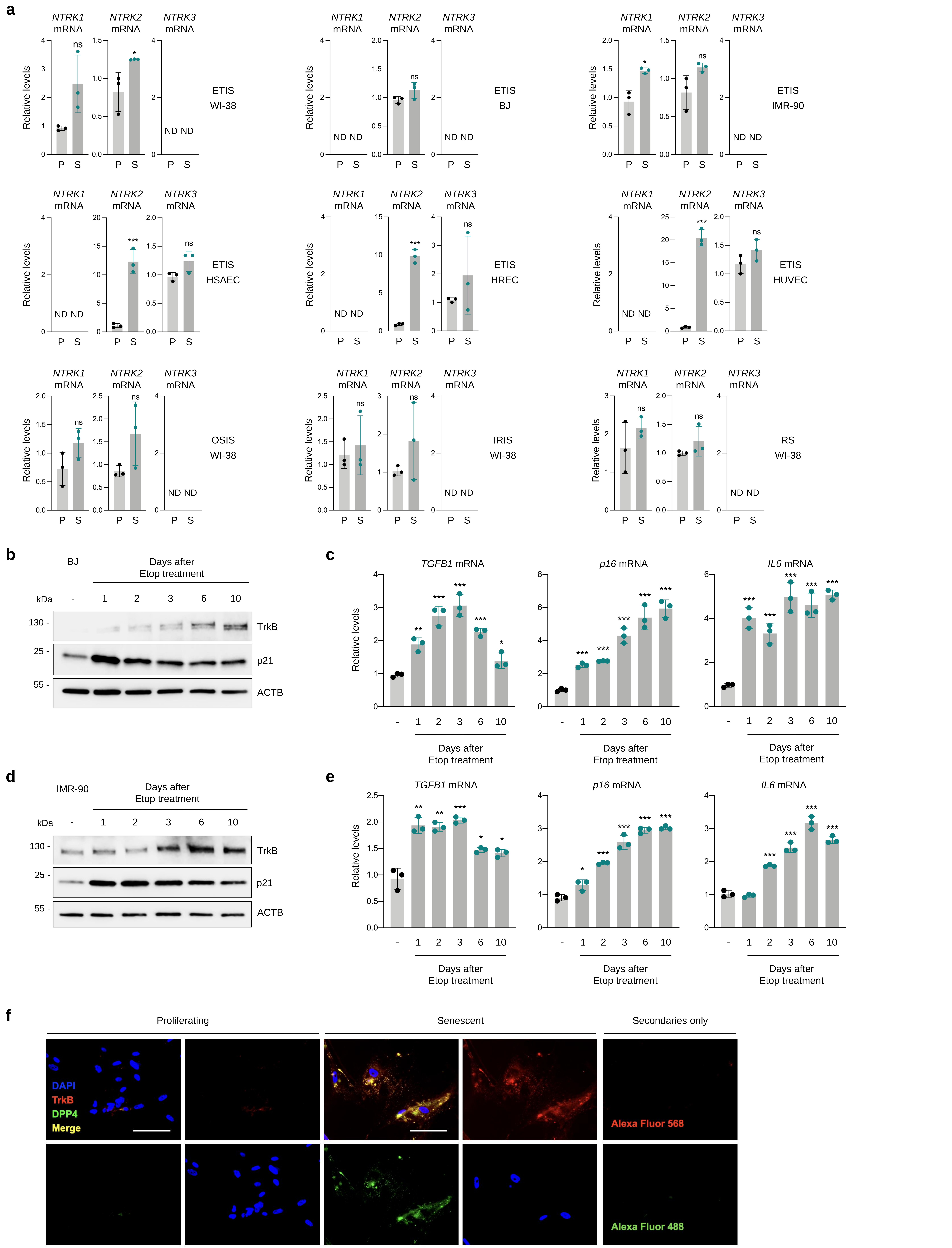

Supplementary Fig. 3. Extended data on TrkB expression throughout senescence. **a** RT-qPCR analysis of mRNAs encoding TrkA, TrkB, TrkC (*NTRK1*, *NTRK2*, *NTRK3* mRNAs, respectively) in different models of senescence (etoposide-induced senescence, ETIS; oxidative stress-induced senescence, OSIS; ionizing radiation-induced senescence, IRIS; replicative senescence, RS) in the indicated cell types. ND, not detected. **b, c** Representative western blot analysis of the levels of TrkB, p21, and loading control ACTB (b) and RT-qPCR measurement of *TGBF1*, *p16*, and *IL6* mRNAs (c) at the indicated days after treating BJ fibroblasts with 25 µM etoposide (Etop) to induce senescence. **d, e** Representative western blot analysis of the levels of TrkB, p21, and loading control ACTB (d) and RT-qPCR assessment of *TGBF1*, *p16*, and *IL6* mRNAs (e) at the indicated days after treating IMR-90 fibroblasts with 50 µM etoposide to induce senescence. **f** Immunofluorescence micrographs showing cells positive for TrkB (red), DPP4 (green), and merged signals (orange/yellow) in proliferating or etoposide-induced senescent WI-38 using non-permeabilizing conditions for detection. Specific staining was confirmed by absent signal in immunofluorescence assays performed with secondary antibodies only. Scale bar, 100 µm. Graphs in a, c, e display the mean values ±SD of n=3 experiments; significance (*p < 0.05, **p < 0.01, ***p < 0.001) was determined by using two-tailed Student's t-test.

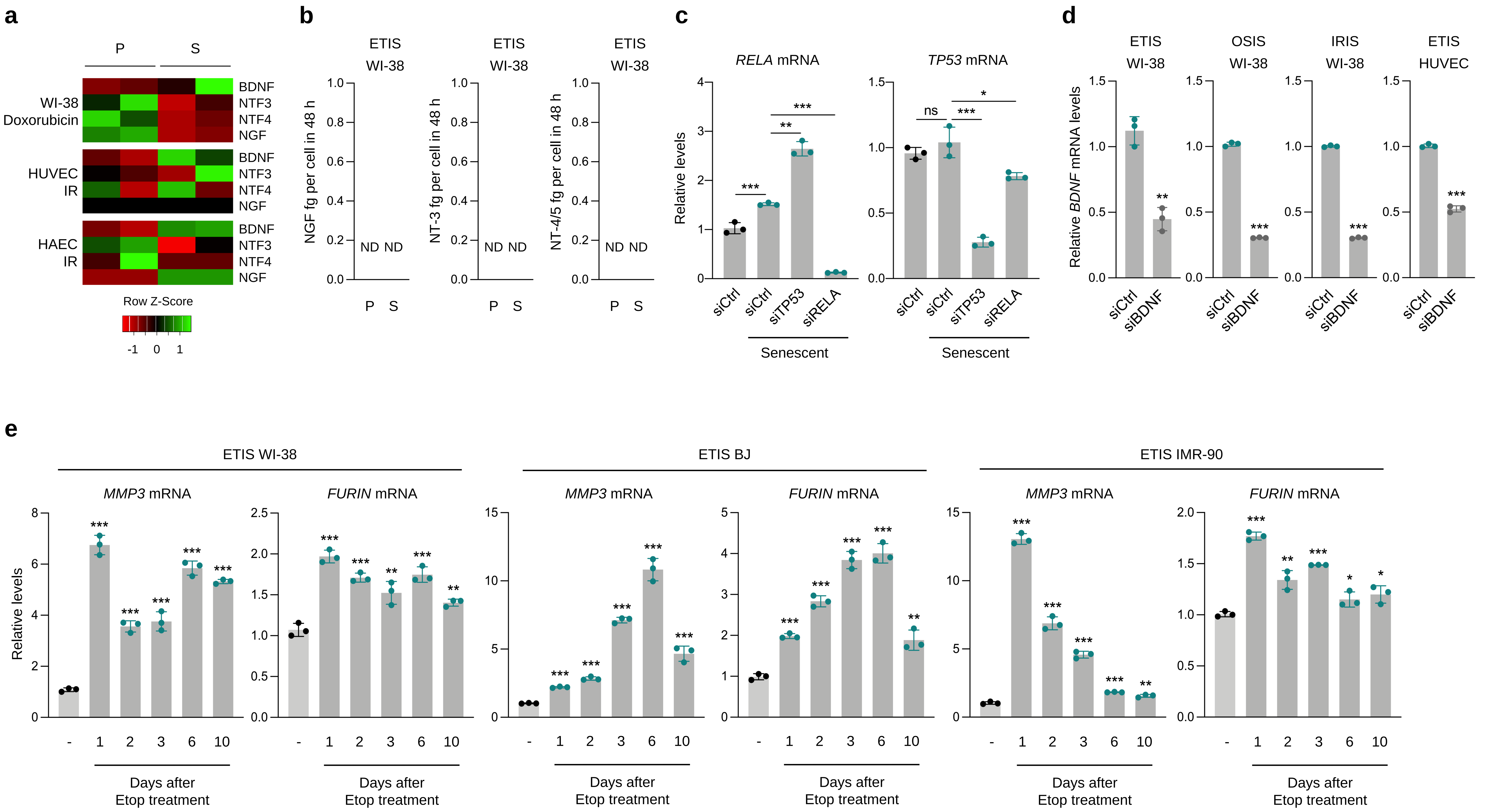

Supplementary Fig. 4. Extended data on BDNF as a previously unidentified SASP factor. **a** Heat maps displaying the relative levels of mRNAs encoding the indicated neurotrophins in several models of cellular senescence obtained from GSE130727. Expression values are shown by row Z-score values and represented as a graded green-black-red color scale. **b** ELISA measurement of the levels of NGF, NT-3, and NT-4/5 in conditioned media collected for 48 h from proliferating and senescent WI-38, BJ, and IMR-90 fibroblasts. ND, not detected. **c, d** Confirmation of silencing of *TP53*, *RELA*, and *BDNF* mRNAs in etoposide-induced senescent WI-38 cells transfected with siTP53 and siRELA (c); and in WI-38 cells undergoing ETIS, OSIS, IRIS and HUVECs undergoing ETIS, each transfected with siBDNF siRNA (d). Cells were transfected and the indicated treatments were started 24 h later. The levels of the different mRNAs were assessed at day 10 into senescence. **e** RT-qPCR analysis of *MMP3* and *FURIN* mRNAs in the indicated models of senescence at different times throughout senescence implementation. Graphs in b-e display the mean values ± SD of n=3 experiments; significance (*p < 0.05, **p < 0.01, ***p < 0.001) was determined by using two-tailed Student's t-test.

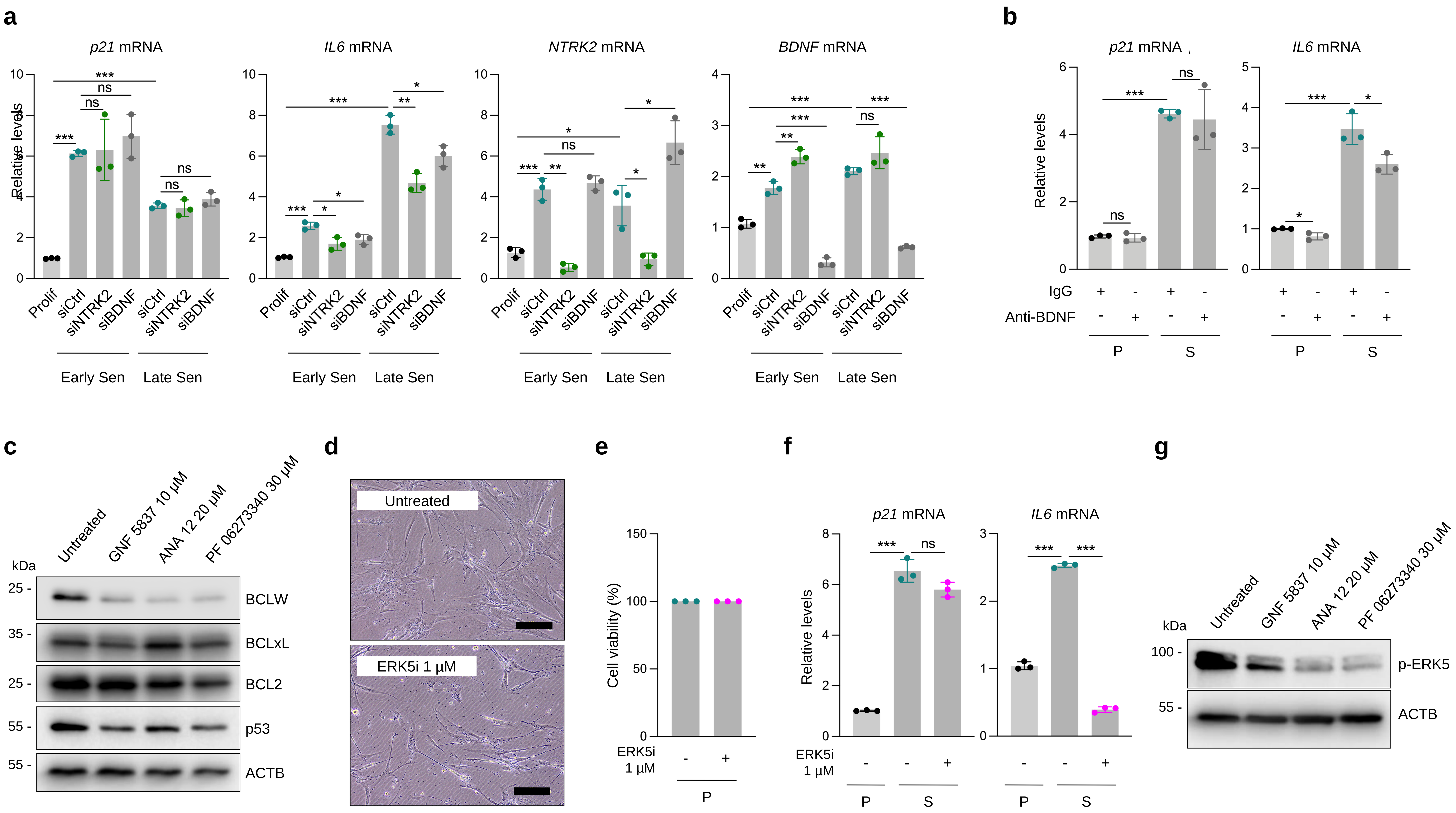

Supplementary Fig. 5. Extended data on signaling through BDNF-TrkB-ERK5-BCL2L2 in senescent cells. **a** RT-qPCR analysis of the levels of *p21*, *IL6*, *NTRK2*, and *BDNF* mRNAs at both early and late senescence (2 and 8 days after etoposide treatment, respectively), in WI-38 fibroblasts transfected with the indicated siRNAs. **b** RT-qPCR analysis of the levels of *p21* and *IL6* mRNAs in WI-38 fibroblasts that were proliferating (P) or rendered senescent (S) after etoposide treatment for 8 days, then treated with IgG or BDNF-blocking antibodies (4 µg/ml) for 48 h. **c** Western blot analysis of the levels of BCLW, BCLxL, BCL2, and p53 in lysates from WI-38 cells undergoing etoposide-induced senescence (ETIS), treated with the indicated TrkB inhibitors for 48 h. **d** Representative phase-contrast micrographs of senescent WI-38 fibroblasts that were either left untreated or treated with the ERK5 inhibitor (ERK5-in-1, 1 µM) for 48 h. **e** Viability of proliferating WI-38 cells treated with ERK5i (ERK5-in-1, 1 µM) for 48 h. **f** RT-qPCR analysis of the levels of *p21* and *IL6* mRNAs in senescent WI-38 cells that were either untreated or treated with ERK5i (1 µM) for 48 h. Control samples were untreated, proliferating WI-38 fibroblasts. **g** Western blot analysis of the levels of p-ERK5 (T218/Y220) levels and ACTB in the conditions described in (c). Graphs (a-c, e, f), represent the values ± SD from n=3 experiments; significance (*p < 0.05, **p < 0.01, ***p < 0.001) was determined by using two-tailed Student's t-test.

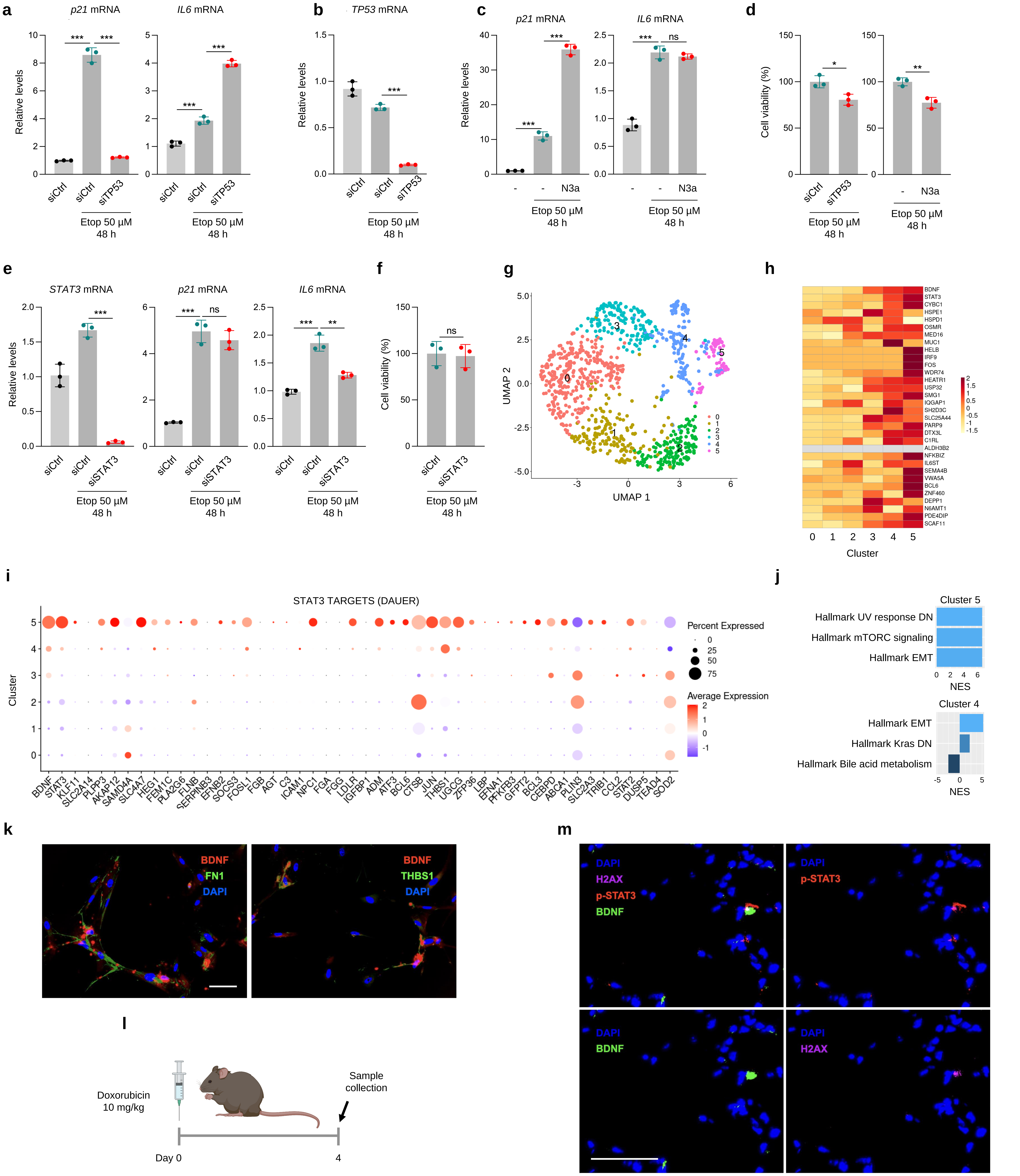

Supplementary Fig. 6. Extended data on BDNF as a discrete marker of survival in cellular senescence. **a** RT-qPCR analysis of the levels of *p21* and *IL6* mRNAs in WI-38 fibroblasts transfected with siCtrl or siTP53 siRNAs and then treated with 50 µM etoposide (Etop) for 48 h. Untreated siCtrl-transfected WI-38 fibroblasts were included as controls. **b** RT-qPCR analysis of *TP53* mRNA in the cultures studied in (a). **c** RT-qPCR analysis of the levels of *p21* and *IL6* mRNAs in WI-38 fibroblasts treated with 50 µM etoposide for 48 h in the presence or absence of the p53-stabilizing compound Nutlin-3a (N3a, 10 µM). Untreated proliferating WI-38 fibroblasts were included as a control group. **d** Assessment of senescent cell viability in the conditions described in (a) and (c) by direct cell counting. **e** RT-qPCR analysis of the levels of *STAT3*, *p21* and *IL6* mRNAs in WI-38 fibroblasts transfected with siCtrl or siSTAT3 siRNAs, then treated with 50 µM etoposide for 48 h. **f** Viability of WI-38 cells transfected with siCtrl or siSTAT3 siRNAs, then treated for 48 h with 50 µM etoposide. **g** UMAP plot of single-cell transcriptomes of WI-38 cells subjected to etoposide-induced senescent (ETIS), showing different clusters defined for the subsequent analyses. **h** Heat map showing association of STAT3 ChIP targets obtained from the ChIP-ATLAS database with the gradient of BDNF expression observed from cluster 0 to cluster 5. **i** Plot displaying the association of the different clusters set in (g) with STAT3 targets obtained from 'STAT3 TARGETS DAUER' GSEA gene set. Dot size and color represent the percentage of cells expressing a transcript and the average expression value, respectively. **j** Normal enrichment score values observed for clusters 5 and 4 in GSEA analysis for the indicated gene sets. **k** Immunofluorescence staining of either FN1 or THBS1 (green), BDNF (red), and DAPI (blue) in ETIS WI-38 cells. White scale bar represents 100 µm. **l** Schematic depicting a model of doxorubicin-induced senescence in mice (created using BioRender). Samples were collected 4 days after treating with 10 mg/kg doxorubicin. **m** Immunoflourescence staining of lungs from samples described in (l) displaying H2AX (purple), p-STAT3 (Y705) (red), BDNF (green), and DAPI. Scale bar, 50 µm. Values shown in a-f are values ± SD; significance (*p < 0.05, **p < 0.01, ***p < 0.001) was determined by using two-tailed Student's t-test.

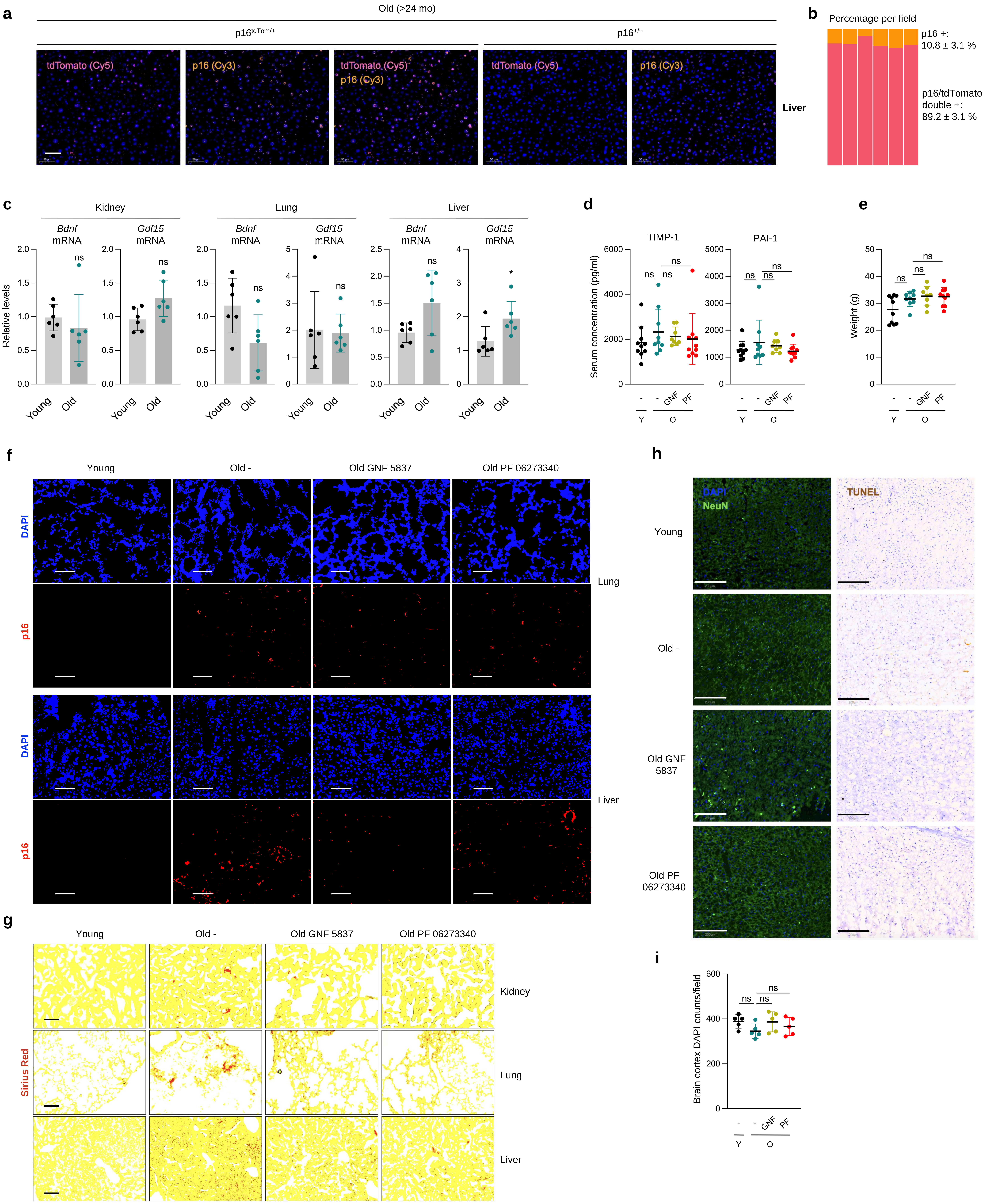

Supplementary Fig. 7. Extended data on Trk inhibitors reducing cellular senescence markers in old mice. **a** Immunofluorescence images from p16-tdTomato knockin mouse [>24 months old (mo)] liver in which the indicated antibodies were used. Controls without the knockin allele were included to assess background signals. **b** Quantification of the signals shown in (a) in 5 different fields from n=2 mice. **c** Levels of *Bdnf* mRNA and *Gdf15* mRNA as measured by RT-qPCR analysis, and normalized to the levels of *Actb* mRNA, in total RNA prepared from kidney, lung, and liver from Young (3 mo) and Old (24 mo) mice; the graphs reflect individual data points measured from different mice. **d** Serum concentrations of TIMP-1 and PAI-1 in the four mouse groups shown, analyzed using a Bioplex instrument. **e** Weights of the mice included in the indicated groups at the end of the experiments (Y are 3 mo, O are 24 mo). **f** Representative p16 immunofluorescent micrographs from liver and lung in the indicated groups. **g** Sirius Red staining representative images from the indicated groups and tissues. **h** Neuronal marker NeuN immunofluorescence (left) and TUNEL assay (right) in brain cortex samples from the indicated groups. **i** Quantification of the DAPI counts per field in brain cortex from n=5 mice per group of the indicated conditions. Scale bars, 200 µm in all the images except in (a). Values shown in c-e, and i are individual data points with the values ± SD; significance (*p < 0.05, **p < 0.01, ***p < 0.001) was determined by using two-tailed Student's t-test; 'ns', not significant.
