# Supplementary material for: A BDNF-TrkB autocrine loop enhances senescent cell viability
Source: Nat Commun. 2022 Oct 20;13:6228. doi: 10.1038/s41467-022-33709-8 (PMC9585019; doi:10.1038/s41467-022-33709-8)
Supplement: Supplementary file 3 — Reporting Summary [file 41467_2022_33709_MOESM3_ESM.pdf]

nature portfolio

Corresponding author(s): NCOMMS-22-01515

Last updated by author(s): 2022-25-1

Reporting Summary

Nature Portfolio wishes to improve the reproducibility of the work that we publish. This form provides structure for consistency and transparency in reporting. For further information on Nature Portfolio policies, see our Editorial Policies and the Editorial Policy Checklist.

Statistics

For all statistical analyses, confirm that the following items are present in the figure legend, table legend, main text, or Methods section.

n/a	Confirmed	
☐	☒	The exact sample size (*n*) for each experimental group/condition, given as a discrete number and unit of measurement
☐	☒	A statement on whether measurements were taken from distinct samples or whether the same sample was measured repeatedly
☐	☒	The statistical test(s) used AND whether they are one- or two-sided<br>*Only common tests should be described solely by name; describe more complex techniques in the Methods section.*
☒	☐	A description of all covariates tested
☒	☐	A description of any assumptions or corrections, such as tests of normality and adjustment for multiple comparisons
☐	☒	A full description of the statistical parameters including central tendency (e.g. means) or other basic estimates (e.g. regression coefficient) AND variation (e.g. standard deviation) or associated estimates of uncertainty (e.g. confidence intervals)
☒	☐	For null hypothesis testing, the test statistic (e.g. *F*, *t*, *r*) with confidence intervals, effect sizes, degrees of freedom and *P* value noted<br>*Give P values as exact values whenever suitable.*
☒	☐	For Bayesian analysis, information on the choice of priors and Markov chain Monte Carlo settings
☒	☐	For hierarchical and complex designs, identification of the appropriate level for tests and full reporting of outcomes
☒	☐	Estimates of effect sizes (e.g. Cohen's *d*, Pearson's *r*), indicating how they were calculated

Our web collection on statistics for biologists contains articles on many of the points above.

Software and code

Policy information about availability of computer code

Data collection	Sequencing BCL files were de-multiplexed and converted to FASTQ files using bcl2fastq program (v2.20.0.422)
Data analysis	GSEA (v 7.4) (https://www.gsea-msigdb.org/gsea/index.jsp)<br>ImageJ (https://imagej.nih.gov/ij/)

For manuscripts utilizing custom algorithms or software that are central to the research but not yet described in published literature, software must be made available to editors and reviewers. We strongly encourage code deposition in a community repository (e.g. GitHub). See the Nature Portfolio guidelines for submitting code & software for further information.

Data

Policy information about availability of data

All manuscripts must include a data availability statement. This statement should provide the following information, where applicable:

- Accession codes, unique identifiers, or web links for publicly available datasets
- A description of any restrictions on data availability
- For clinical datasets or third party data, please ensure that the statement adheres to our policy

RNA-seq data are deposited in GSE190998 (token crqxgaculzcxnid). These data have been analyzed and displayed in Fig 5d.

Field-specific reporting

Please select the one below that is the best fit for your research. If you are not sure, read the appropriate sections before making your selection.

☒ Life sciences ☐ Behavioural & social sciences ☐ Ecological, evolutionary & environmental sciences

For a reference copy of the document with all sections, see nature.com/documents/nr-reporting-summary-flat.pdf

Life sciences study design

All studies must disclose on these points even when the disclosure is negative.

Sample size	No sample size calculations were performed for the in vitro work. We utilized at least 3 independent experiments for each of the experiments described in this report. For in vivo, we determined that at least n=8 was sufficient to show the differences among experimental groups while using a reduced number of animals.
Data exclusions	No data were excluded from this work.
Replication	Experiments were performed at least 3 times independently.
Randomization	In vitro experiments were performed independently while keeping all the groups tested at each experiment together. Treatments performed in mice were given randomly and always representing all the experimental groups within the different litters or cages utilized.
Blinding	Blinding was carried out for the analysis of imaging techniques performed in vivo. Samples were blinded by IDs during image analysis.

Reporting for specific materials, systems and methods

We require information from authors about some types of materials, experimental systems and methods used in many studies. Here, indicate whether each material, system or method listed is relevant to your study. If you are not sure if a list item applies to your research, read the appropriate section before selecting a response.

Materials & experimental systems

n/a	Involved in the study
☐	☒ Antibodies
☐	☒ Eukaryotic cell lines
☒	☐ Palaeontology and archaeology
☐	☒ Animals and other organisms
☒	☐ Human research participants
☒	☐ Clinical data
☒	☐ Dual use research of concern

Methods

n/a	Involved in the study
☒	☐ ChIP-seq
☒	☐ Flow cytometry
☒	☐ MRI-based neuroimaging

Antibodies

Antibodies used	phosphorylated p38 MAPK (T180/Y182) (Biolegend, Ref. 903501), phosphorylated SAPK/JNK (T183/Y185) (81E11) (Cell Signaling, Ref. 4668S), phosphorylated ERK1/2 (T202/Y204) (Biolegend, 675502), phosphorylated AKT (Ser473) (Cell Signaling, 4060S), phosphorylated STAT3 (Y705) (D3A7) XP® (Cell Signaling, 9145S), p53 (DO-1) (Cell Signaling, 18032S), ACTB (β-Actin C4) (Santa Cruz Biotechnology, sc47778), and BCL2L2/BCL-w (Cell Signaling, 2724S), phosphorylated PKCα/β II (Thr638/641) (Cell Signaling, 9375S), phosphorylated ERK5 (Thr218/Tyr220) (Cell Signaling, 3375), p21 (Santa Cruz Biotechnology, sc-53870), TrkA (Cell Signaling, 2510S), BCLxL (54H6) (Cell Signaling, 2764S), BCL2 (D55G8) (Cell Signaling, 4223S), phosphorylated Histone H2A.X (Ser139) (20E3) (Cell Signaling, 9718S), H2AX (ab140498, Abcam), p-STAT3 (Tyr705) (4113, Cell Signaling Technology), NeuN (24307, Cell Signaling Technology), FN1, (Abcam (ab2413), THBS1 (Abcam (ab85762)), and TrkB (Santa Cruz Biotechnology, sc136990).
Validation	Well-characterized antibodies were used for this study, most of them displaying several positive reviews on the manufacturer's pages.

Eukaryotic cell lines

Policy information about cell lines

Cell line source(s)	Human IMR-90 (ATCC), WI-38 (Coriell Institute), BJ fibroblasts, HUVEC (Endothelial cells), HSAEC (Lung Small Airway Epithelial primary cells), and HREC (Kidney Epithelial primary cells) (ATCC).
Authentication	The cell lines were not authenticated separately.

Mycoplasma contamination	All cell lines were Mycoplasma-negative.
Commonly misidentified lines (See ICLAC register)	No commonly misidentified cell lines were used in the study.

Animals and other organisms

Policy information about studies involving animals; ARRIVE guidelines recommended for reporting animal research

Laboratory animals	C57BL/6J mice (Stock #000664) 3 months old or 18 months old were imported from the Jackson Laboratory (Bar Harbor, ME), and housed in the animal facility in NIA. Both males and females were used.
Wild animals	The study did not involve wild animals.
Field-collected samples	The study did not involve samples collected in the field.
Ethics oversight	The NIA Animal Care and Use Committee (ACUC) provided guidance and oversight for the animal experiments.

Note that full information on the approval of the study protocol must also be provided in the manuscript.
